# Fingerprint resampling: A generic method for efficient resampling

**DOI:** 10.1038/srep16970

**Published:** 2015-11-24

**Authors:** Merijn Mestdagh, Stijn Verdonck, Kevin Duisters, Francis Tuerlinckx

**Affiliations:** 1 University of Leuven, Oude Markt 13, 3000 Leuven, Belgium.; 2 Leiden University, Rapenburg 70, 2311 EZ Leiden, Netherlands.

## Abstract

In resampling methods, such as bootstrapping or cross validation, a very similar computational problem (usually an optimization procedure) is solved over and over again for a set of very similar data sets. If it is computationally burdensome to solve this computational problem once, the whole resampling method can become unfeasible. However, because the computational problems and data sets are so similar, the speed of the resampling method may be increased by taking advantage of these similarities in method and data. As a generic solution, we propose to learn the relation between the resampled data sets and their corresponding optima. Using this learned knowledge, we are then able to predict the optima associated with new resampled data sets. First, these predicted optima are used as starting values for the optimization process. Once the predictions become accurate enough, the optimization process may even be omitted completely, thereby greatly decreasing the computational burden. The suggested method is validated using two simple problems (where the results can be verified analytically) and two real-life problems (i.e., the bootstrap of a mixed model and a generalized extreme value distribution). The proposed method led on average to a tenfold increase in speed of the resampling method.

Resampling methods refer to a variety of different procedures in which a Monte Carlo method is used to create a large number of resampled versions of the original data. Well known examples include bootstrapping^1^, permutation tests and various sorts of cross-validation. Commonly, resampling methods are used to assess the uncertainty of an estimator, to estimate tail area probabilities for hypothesis testing, to assess prediction errors or to do model selection^2^.

Broadly speaking, all resampling methods follow the same general scheme. First, the original data set is resampled multiple times. Resampling can be done directly on the original data set or through a statistical model that is first fitted to the data. In a next step, a model is estimated for the resampled data. Finally, the results of the estimated models are combined to calculate, for example, the uncertainty of an estimator of interest (e.g., confidence intervals and predictive validity obtained using bootstrapping and cross-validation, respectively).

A major reason for the popularity of these resampling methods stems from their flexibility^3^,^4^,^5^,^6^,^7^. They allow a researcher to compute uncertainty assessments or hypothesis tests in situations where no analytical solution is available or where some assumptions of the analytical method are not justified (e.g., deviations from normality).

Unfortunately, even in these times of abundant computer power, a main disadvantage of these resampling methods is the computational burden. This computational burden is specifically present when for every regenerated data set, an iterative method is needed to maximize a likelihood or to minimize a squared error. If such an optimization is costly, the resampling method may become practically unfeasible.

As an illustration of the problem, one may think of the calculation of a bootstrap based confidence interval (CI) for a variance component in a linear mixed model^8^. Linear mixed models are commonly used in the life sciences^9^,^10^,^11^,^12^,^13^. More specifically, we consider a data set and model described in Samuh *et al*^14^. A sample of 129 depressed individuals is measured 71 times on average (in total there are about 9115 time points) regarding their momentary sadness. The design is unbalanced because not all individuals have the same number of measurements. A longitudinal linear mixed model (see Verbeke and Molenberghs^8^) with the previous emotional state as a predictor is fitted to the data. Both intercept and slope are assumed to vary randomly across patients.

Our prime interest for these data is in the variance components (i.e., the estimates of the between patient variances regarding intercept and slope). Current analytical procedures for computing such a variance component CI are not accurate enough and therefore a bootstrap based CI is pursued as an alternative^15^,^16^,^17^. If we aim at 2000 bootstrap replications, then repeatedly fitting the linear mixed model would take more than four hours using one core on an Intel Core i7-3770 with 3.4 GHz computer. Such computation times may prohibit the routine use of the bootstrap by applied researchers.

Together with the rise in popularity of resampling methods, there have been continued efforts to alleviate the computational burden of these methods. For example, Efron and Bradley proposed to decrease the number of bootstraps in favorable cases^18^. They improved the post processing of the bootstrap estimates, increasing the accuracy for the same number of bootstrap estimates computed. However, most efforts are concentrated on decreasing the computational burden of the model fit on a single resampled data set by, for example, reducing the number of iterations in the optimization method^9^,^10^,^11^,^12^,^13^,^14^,^15^,^16^,^17^,^18^,^19^,^20^,^21^. Another way to speed up the resampling process is to split the estimating function into smaller, more simple parts. Bootstrapping these parts can lead to faster and more accurate bootstrapping^22^. More recent improvements focus on tailor-made solutions for specific problems^23^,^24^,^25^. Unfortunately, none of these methods are generic and easily convertible to other situations.

In this paper, we will propose a generic procedure to decrease the computational burden. As a result, resampling methods can be used for problems that are very time-consuming because an iterative optimization is needed. The key idea of our method is that for all resampled data, a similar task has to be performed because the same model has to be fitted to very similar data. Consequently, when this task is performed repeatedly, we may learn from the past and use this knowledge for future problems. This core idea is very simple and can be applied to any resampling technique.

More specifically, the optimization algorithm needs an initial (or starting) value for each resampled data set. By improving the quality of this initial value, the optimization problem can be significantly speeded up because the closer the initial value to the real optimum, the less iterations the algorithm needs to converge. The method we propose will use information from previous fits to considerably improve the starting points for subsequent problems. In addition, if the initial value is accurate enough, the optimization process may even be omitted.

## Results

### An optimal choice of the starting values

Two simple examples (involving the exponential distribution and a simple linear regression) will be used to motivate and introduce our method on how to choose the starting values of numerical optimization algorithms in a resampling context optimally. In both examples, there are two artificial aspects that make them simple examples. First, we will make use of the bootstrap in both situations, but the simple and well-understood properties of these models make the use of the bootstrap unnecessary. Second, our proposed method will only provide an advantage if the estimation process makes use of a numerical optimization algorithm. However, for both examples, closed-form estimators are available. Therefore, we will nevertheless make use of an iterative optimization algorithm and act as if the analytical solution is not known. An advantage of working with such simple models with available closed-form expressions is that some results can be checked analytically.

In a first example, we will bootstrap an exponential distribution. The loglikelihood of such an exponential distribution, with data set 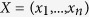 is given by




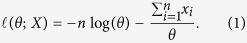




For a positive 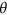, the maximum of this equation is found by setting its first derivative to zero:




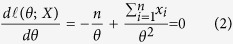




and solving for 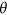:




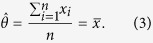




**Figure 1:**
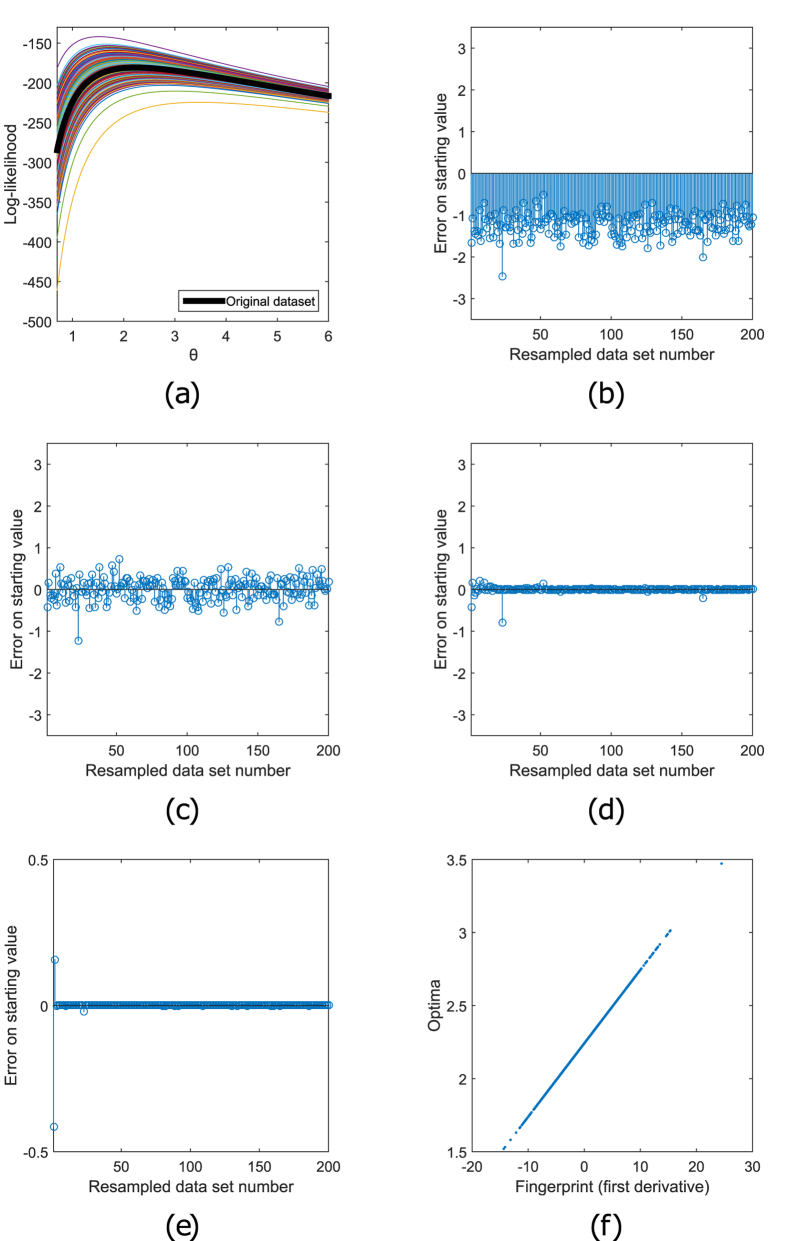
Choosing optimal starting values for an exponential model. In (**a**) the loglikelihood functions of the exponential distribution for the original data set and 200 resampled data sets are shown. The optima (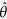) of all these loglikelihood functions need to be found using an iterative optimization proces. Each optimization needs a starting value 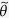. In (**b–e**), the horizontal axis shows the resampled data set number 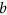 and the vertical axis the initial errors on the starting values 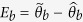. In (**b**), option 1 is used to choose this starting value (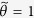). All initial errors are negative. (**c**) shows that this bias of the starting values can be avoided by using option 2. The original optimum, 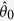 is more central compared to the other optima. In (**d**) it is shown that this error decreases further when the nearest neighbor variant of option 3 is used. In (**e**), where the interpolation variant of option 3 is used, the error almost vanishes. Using first order derivatives as fingerprint, the optima are easy to predict. In (**f**) it is shown that there is a linear relation between fingerprints 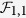 to 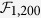 and optima 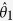 to 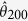.

The resulting optimum is the sample average, 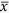. The analytical solution will only be used to verify our findings and to provide a better insight in the workings of our method. Let us now simulate a data set, denoted as the original data 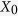, with *θ* = 2, 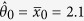 and *n* = 100. For the bootstrapping process, the data sets are non-parametrically resampled *B* = 200 times 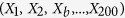. The loglikelihoods of the original data set and 200 resampled data sets are shown in Fig. 1a.

For every resampled data set 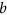, a loglikelihood needs to be optimized to find its maximum, 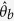. In an iterative estimation procedure, this means that for every resampled data set 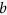, we also need a starting value, 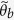. (In the remainder of the paper, we will denote starting values by a tilde.) The closer the starting value to the optimum, the faster the optimization algorithm will converge to the optimum. The difference between the starting value and the optimum for a bootstrap sample, 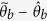, will be called the initial error and denoted as 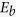.

Let us now consider in turn three options we have when choosing these starting values for the 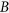 bootstrap samples.

#### Option 1: A naive starting value

In an extreme situation, the researcher barely knows anything about the problem at hand and chooses a common naive starting value, for example, 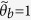 (for all 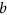). The initial error corresponding with this naive starting value, 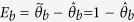, is shown in Fig. 1b for all resampled data sets. In this case, the starting value is clearly an underestimation of the optimum, and the error is always negative.

#### Option 2: The original optimum 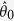 as a starting point

An easy improvement can be made at this point by taking the original optimum as a starting value:




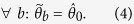




**Figure 2:**
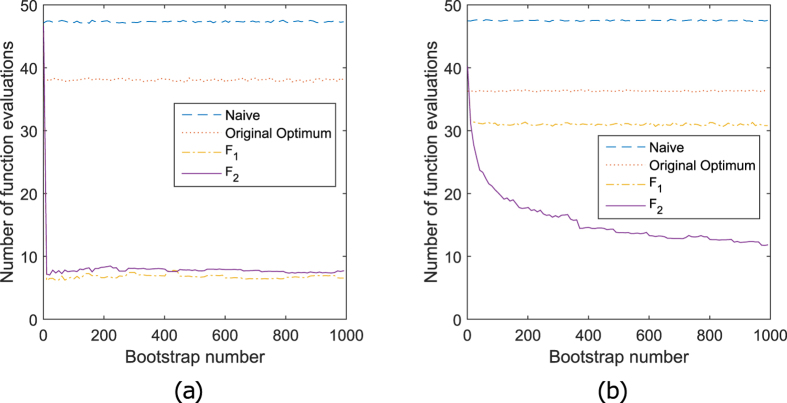
Number of function evaluations per bootstrap for the two simple problems. For both simple problems, the exponential distribution and the linear regression, 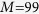 synthetic data sets are simulated. For each simulated data set, 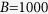 bootstrap data sets are generated. For each bootstrap sample, the optimal parameter value is computed through the iterative Nelder-Mead algorithm. The horizontal axis shows the resampled data set number and the vertical the number of function evaluations needed by the Nelder-Mead algorithm, averaged over the 99 synthetic data sets. Option 1 (naive starting value) and option 2 (original optimum as starting value) use the same starting value for every resampled data set. The optimization speed does not change. Both panels also show two interpolation variants of option 3. Once with first order derivatives (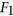 variant) and once with first and second order derivatives (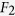 variant). In (**a**) the results for the exponential distribution are shown. The linear relation between the first fingerprint and the optimum (Fig. 9) is easily learned, instantly leading to fast optimizations in variants 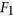 and 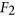. In (**b**) the results for the linear regression are shown. Only when also the second derivative is included in the fingerprint (variant 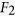), the mean number of function evaluations decreases considerably, relative to option 1 and 2. The decrease in the number of function evaluations is not as fast as in the exponential distribution. The reason is that the relation is nonlinear and more difficult to learn. When more and more resampled data sets are processed, the predictions improve and the number of function evaluations needed per optimization decreases.

The original optimum, 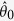, is the optimum that belongs to the fit of the original data set 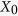 and is typically computed before the resampling process anyway. All the subsequent regenerated data sets, 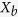, are related to this original data set. The original data set can be considered central. As a consequence, it is likely that also 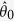 is central to the optima of the resampled data sets, as is the case in Fig. 1a. This leads to a much less biased starting value (Fig. 1c) and a faster optimization process (Fig. 2a).

#### Option 3: Learn from the previously resampled data sets

The starting value can be improved upon further. The same function (Eq. 1), based on very similar data sets (all resampled from 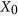), needs to be optimized over and over again. As a result, all the loglikelihoods look very similar, as shown in Fig. 1a.

The core of our method lies in the fact that we try to learn from the past. When one needs to find the estimate 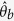 for resampled data set 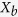, the optima belonging to all previous data sets, 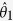 to 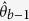, are already found. If 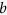 goes to infinity, the optimum of the same data set will already be computed. A better starting value cannot be wished for.

In the more realistic setting of a finite 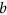, 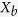 will not be in the set of 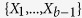. There will however be some data sets that are more similar to 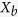 than others. One can search now for the most similar data set. Assume 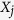 is the most similar data set to 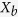. The optimum 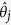 of this similar data set will probably be a good prediction of the optimum belonging to data set 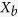. We can take this prediction as a starting value, 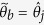. One can call this a nearest neighbor prediction:




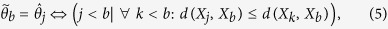




with *d*(*X*_*j*_, *X*_*b*_) a distance between data sets 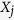 and 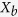. If 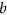 gets larger, more and more resampled data sets are fitted leading to a larger pool of previous data sets to choose from. The chances of finding a very similar data set become larger, leading to better starting values.

Although intuitive to grasp, measuring the distance between raw data sets is often not very easy. A better strategy is to first summarize the resampled data sets into properties (or statistics) that carry information about the optima. Such summary statistics will be called *fingerprints*. A fingerprint 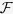 is a statistic or function that takes a resampled data set as input. The idea is that these statistics provide direct information on the optimum that we look for. In general, we propose to use derivative-based fingerprints. In this exponential distribution example, the first derivative of the loglikelihood for the resampled data set evaluated in the original optimum will suffice. Formally, the fingerprint for the resampled data 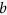 is defined as:




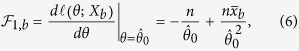




with 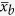 being the sample average of resampled data set 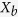. Here the first index of 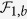 refers to the fact that the first order derivative is used.

Once the derivative-based fingerprints ℱ are computed, we can just take the squared Euclidean distance between them to create a distance measure between the resampled data sets: 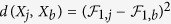. In Fig. 1d the initial errors 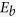 in the starting values computed using Equations 5 and 6 are shown. It is shown that the errors become on average considerably smaller when the number of fitted bootstrap samples increases.

A disadvantage of the nearest neighbor approach is that when a new fingerprint 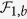 becomes available, only the nearest neighbors to this new fingerprint determine the newly assigned starting point 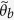. However, we can improve on this practice by using a clever interpolation method. We want to model the relation between the already computed fingerprints 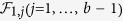 and their corresponding optima 
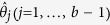
. This relation can then be used to make a prediction and to associate a good starting point 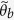 to the new fingerprint 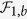.

Because we do not know much about the relation we want to model in general, we choose an interpolation method that is capable of capturing nonlinear relations. Examples of such methods are least squares support vector machines (LS-SVM)^26^,^27^ or multivariate adaptive regression splines (MARS)^28^. We will show only the results obtained by using LS-SVM, however similar results were obtained by using MARS.

The goal of the nonlinear interpolators is to learn the relation 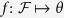 based on the couples 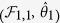 to 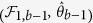. The estimated relation can be denoted as 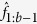 (where the index indicates that the first 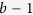 data sets are used). In a next step, the optimum of resampled data set 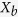 can be predicted:




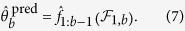




The predicted optimum will then serve as the improved starting point for the resampled data 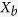:




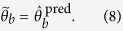




Once again, the predictions will become better when the pool of couples of fingerprints and optima to learn the relation 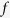 from, grows larger. The errors of the starting values computed with this variant, using LS-SVM, are shown in Fig. 1e. This variant, using the interpolation method in combination with first order derivatives for the fingerprint will be called the 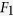 variant. In the remainder of this paper, we will not discuss the nearest neighbor variant anymore and directly consider this interpolation method as it gives much better results.

In fact, after two bootstrap samples, the predicted optimum actually coincides with the true optimum for the resampled data set. Hence, the interpolation method can, based on the fingerprint, almost exactly predict the optimum. This is not a surprise because in this exceptionally easy toy example, there exists an exact functional relation between the fingerprint and optimum. This can be seen when one combines Equation 6 and the analytical solution of the maximum likelihood estimation (Eq. 3):




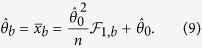




Because of the linearity of this relation, only a set of two training points (Fig. 1e) is enough to create a model that delivers almost perfect starting values, instantly leading to fast optimizations (Fig. 2a). The relation between the fingerprints and the optima for all the resampled data sets is shown in Fig. 1f.

**Figure 3:**
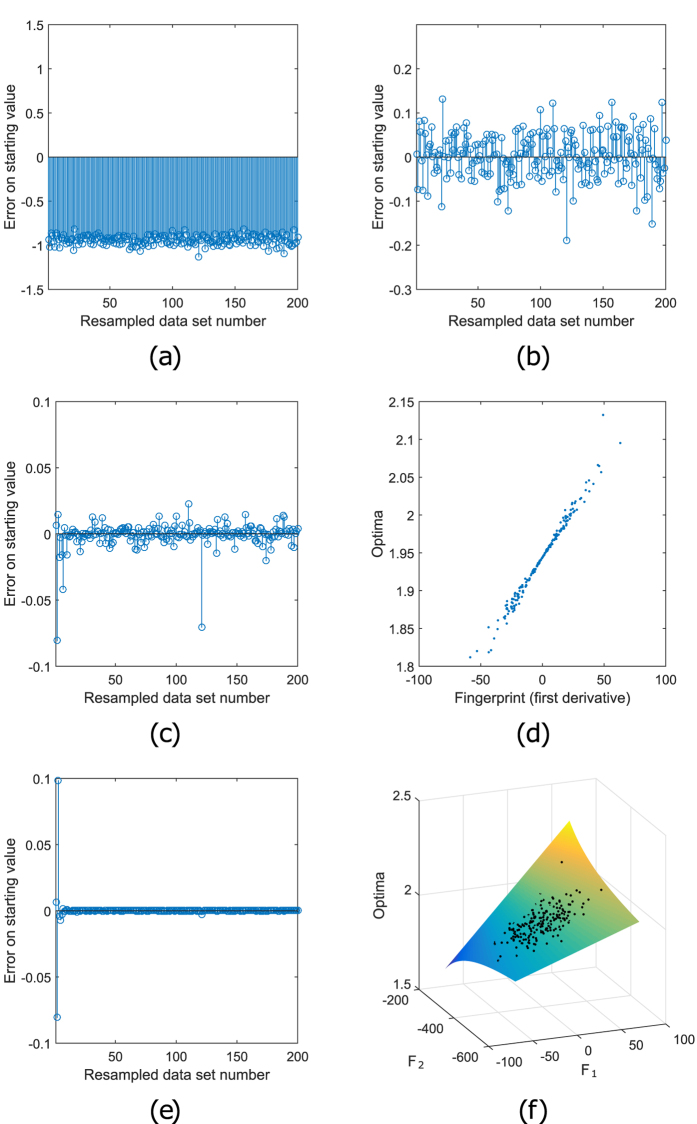
Choosing optimal starting values for a linear regression. A linear regression data set with estimated slope (the original optimum), 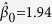, is non-parametrically resampled 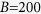 times. In this figure, the possible choices of the starting values for the optimization of the loglikelihood of these resample data sets are compared in (**a–c**) and (**e**). In all those panels the horizontal axis shows the resampled data set number 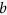 and the vertical axis the initial errors on the starting values 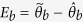. In (**a**), a naive starting value is chosen (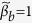, option 1). All errors are negative. In (**b**), the original optimum is chosen as starting value (option 2) which removes the bias. In (**c**), the results of the interpolation variant of option 3, using a one dimensional fingerprint (first order derivative) are shown (variant 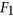). 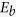 further decreases, but not to machine precision. The reason is shown in (**d**): there is no unique relation between fingerprints 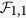 to 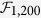 and optima 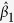 to 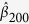. Data sets with the same fingerprint can result in different optima. In (**e**), the initial error 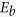 again decreases to zero. Here a two dimensional fingerprint, 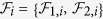, using the first and second-order of differentiation of the loglikelihood, is used (variant 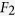). This results in a unique, non-linear relation between the fingerprints and the optima, as is seen in (**f**), showing fingerprints 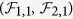 to 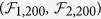 versus optima 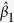 to 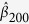.

The options to choose a starting value, described in the previous paragraphs, are generic and can easily be used for other estimation problems. In a second example, they are applied to a linear regression without intercept and with known variance. In Figs 3 and 2b similar results are shown for options 1 and 2. However, for option 3, a one dimensional fingerprint, using only the first order derivative of the loglikelihood, is not sufficient anymore. The accuracy of the starting values improves if also the second order derivative is used, called the 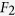 variant. The starting values are now predicted using a two dimensional fingerprint:




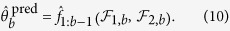




As this new relation is nonlinear (Fig. 3f), it takes more bootstrap samples to be learned, which leads to a more gradual decrease in function evaluations, as shown in Fig. 2b.

### Bootstrap of a mixed model

In the previous paragraphs, we studied our methods by evaluating the number of function evaluations to find the optimum. However, the purpose of a resampling method such as the bootstrap is usually to compute an uncertainty measure (e.g., the standard error of an estimate or a confidence interval). In this result section, we will estimate the lower bound of a 95% confidence interval using the percentile bootstrap confidence interval method^29^.

We will apply this bootstrap on a linear mixed model, a model commonly used in the life sciences^9^,^10^,^11^,^12^,^13^. The bootstrap of a linear mixed model is of interest in a variety of areas^14^,^30^,^31^,^32^,^33^,^34^, as inference for its random effects is not straightforward^15^,^16^,^17^.

In this example we will use real-life data from Bringmann *et al*^14^. As stated in the introduction, 129 depressed patients are measured repeatedly 10 times a day for 10 days using the experience sampling method regarding their momentary sadness^35^. As not all patients respond to every measurement, the data are unbalanced and consists of only 9115 time points. For each patient, sadness at timepoint 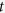 will be predicted by sadness at timepoint 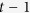. Both the intercept and the slope are assumed to vary across patients. We will use the bootstrap with 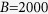 bootstrap iterations to estimate the 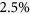 percentile of the variance matrix of these random effects:




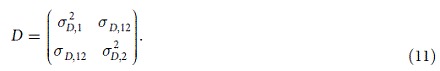




The more bootstrap samples are processed, the higher the accuracy of this 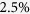 percentiles will become. However, more bootstrap iterations generally means more function evaluations to estimate the mixed model parameters. Hence, there is an intrinsic trade-off between speed (number of function evaluations) and accuracy. Next, we can study how the different options and variants of our method deal with this speed-accuracy trade-off. Option 1, taking naive starting values, will not be considered. All speed ups will be measured against option 2, taking the original optimum as starting value. Moreover, new variants will be introduced.

#### Option 4: Bypass the optimization altogether

In the previous section we showed how better starting values can increase the speed of the optimization process. The end result, the accuracy of the bootstrap, will be exactly the same for all these variations. The different starting values will lead to the same optima (assuming an optimization algorithm fit to find the global optimum). However, when the starting values become better and better, they can become practically indistinguishable from the real optima. Such accurate predictions of new optima make the optimization process superfluous for the associated re-sampled data sets. Skipping the optimization process will lead to an even larger speed increase. Several variants will be considered and they are denoted generically as 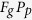. The index 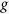 means that partial derivatives of the loglikelihood function up to order 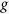 are used for the fingerprint. Finite differences (using an extra number of function evaluations) are used to compute these derivatives. The number 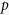 refers to the ratio between the number of resampled data sets for which the optimization is bypassed compared to the number of resampled data sets for which the optimization algorithm is fully used. For example, if 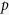 is 3 and 200 bootstrap estimates are found with an optimization algorithm, then 600 predicted estimates are added to create a total set of 800 estimates. However, the total number of estimates never exceeds 2000 (as we have 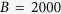 bootstrap iterations). So with the different values of 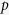, maximum 200 predicted estimates will be added if already 1800 estimates are found with an optimization process. Note that the 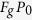 variants are the same as the 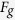 variants of option 3, where the optimization is never bypassed. All different options and variants are summarized in Table 1.

**Figure 4:**
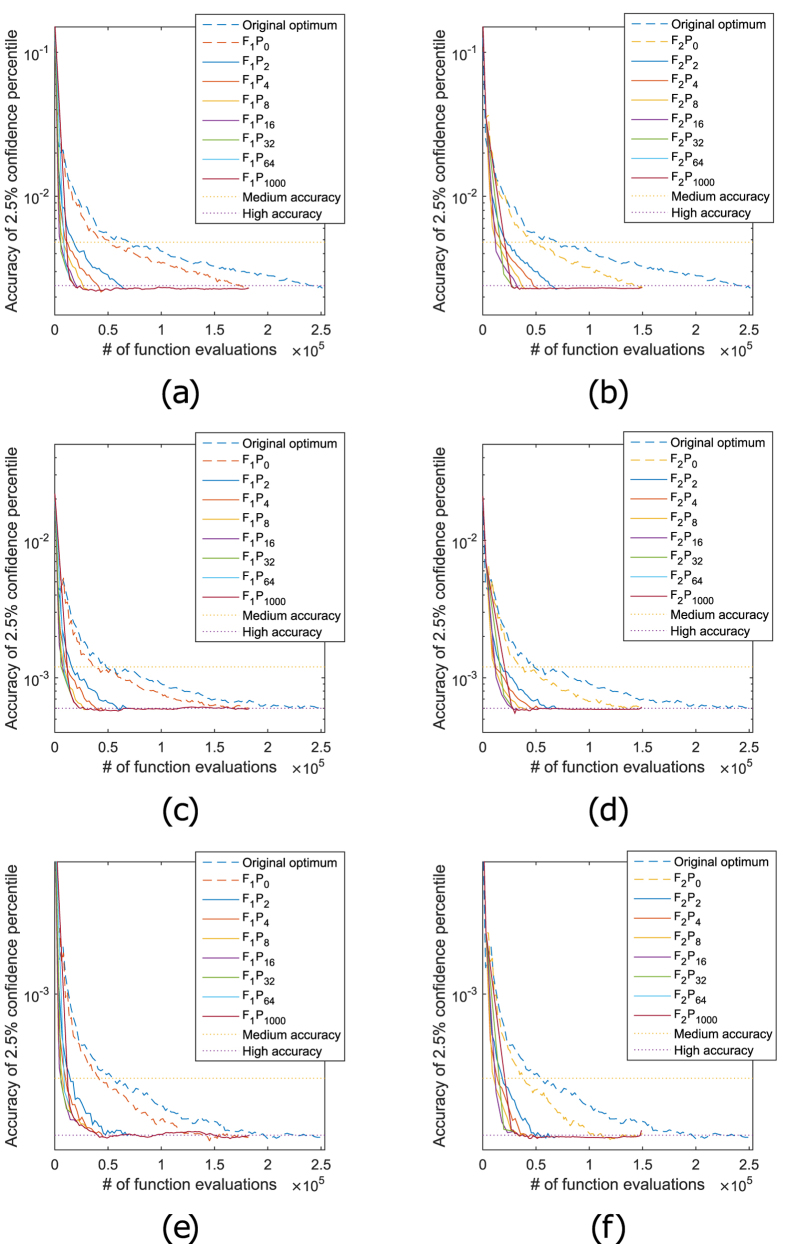
Trade off between accuracy and speed for the bootstrap of a mixed model. 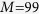
 times, a mixed model is bootstrapped to compute the 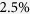 percentile of the random effect variance parameters 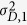, σ_D,12_ and 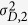. Each bootstrap has 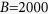 bootstrap iterations. On each panel, the error of this 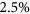 percentile averaged over the 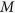 bootstrap processes, measured as described in the method section, is shown on the vertical axis. The average speed, measured in number of function evaluations, is shown on the horizontal axis. On the left panels (in (**a**), (**c**) and (**e**)), the results for variants 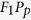 are shown, using only first order derivatives for the fingerprints. On the right panels (in (**b**), (**d**) and (**f**)), derivatives up to order two are used. In (**a**) and (**b**), the bootstrap is applied on the variance parameter of the random intercept 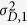. In (**e**) and (**f**), the bootstrap is applied on the variance parameter of the random slope 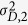. In (**c**) and (**d**) the bootstrap is applied on the covariance parameter, σ_D,12_, of these random effects. The dotted lines show a high accuracy (approximately the lowest error achieved by the original optimum variant) and a medium accuracy (approximately twice this error), as used in Table 2. More function evaluations always lead to a better accuracy (lower error) of the 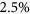 percentiles. It is shown that the various 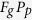 methods reach the same accuracy much faster compared to the original optimum variant.

The resulting speed up is shown in Fig. 4 and Table 2. The accuracy is shown against the number of function evaluations, i.e. the computational burden. Our method can acquire the same accuracy up to 17 times as fast as the original optimum variant. More generally, for all the model parameters over all accuracies, approximately a tenfold speed up can be acquired. Using finite differences, more function evaluations are needed to generate a fingerprint that includes higher order derivatives. To get low quality predictions, a fingerprint of a lower order (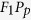) suffices. Therefore, it is faster to use only first order derivatives when only a low accuracy of the 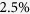 percentile is required. When a higher accuracy of this 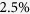 percentile is required, it can be better to use more informative fingerprints, with higher order derivatives, to create better predictions. For parameters 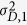 and σ_D,12_ a fingerprint that uses only first order derivatives is enough. For a medium as well as high accuracy, the 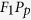 variants outperform the 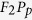 variants. However, for the third parameter, 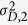, 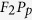 is a better option when a high accuracy is required. The better predictions now outweigh the extra cost of the larger fingerprint. It is also interesting to note that the 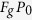 variant always gives the worst speed up. This means that, for this example, it is always advantageous to bypass the optimization process for at least a part of the resampled data sets.

### Bootstrap of a generalized extreme value model

As a second real-life application we will apply fingerprint bootstrapping on a generalized extreme value (GEV) distribution model^36^. Also for the inference of GEV distributions, bootstrapping is a well-used tool^37^,^38^,^39^,^40^. Again we will estimate the lower bound of the 95% confidence interval of the parameters of the GEV distribution.

In De Kooy, The Netherlands, the maximum hourly mean windspeed was measured almost daily between 1906 and 2015^41^. In total 39760 maxima where included. The GEV with one covariate (i.e., date of measurement) will be used to model these maxima. As explained in the method section, the estimation of this model leads to a four dimensional optimization problem, with parameters 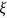 (shape) , 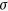 (scale), 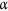 (intercept) and 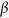 (regression weight).

By counting the number of function evaluations needed to have a medium or high accuracy of the lower bound of the 95% confidence interval, we calculate the speed up of the fingerprint resampling method as shown in Table 3. The speed up of the 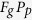 over the original optimum variation is even bigger as with the mixed model. The reason is that for the GEV distribution, using only the first derivatives of the likelihood as fingerprint, leads to sufficiently good prediction accuracies. Therefore, the 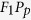 variants can be used for the medium as well as the high accuracy. As the number of function evaluations needed to create the fingerprints of these 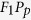 variants is significantly lower, the speed up is bigger. To achieve a high accuracy in the lower bound of the 95% confidence interval, a speed up of about 25 is acquired for all the variables for the 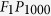 variants. For the 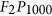 variants again a speed up of about 10 is reached. For the medium accuracies the 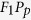 variants lead to speed ups around 10. Again, the 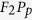 variants do not perform very well when only a medium accuracy is needed. Too much time is needed to compute the detailed fingerprints, while only a medium accuracy is required.

### Effect of parallelization on the speed up

**Figure 5:**
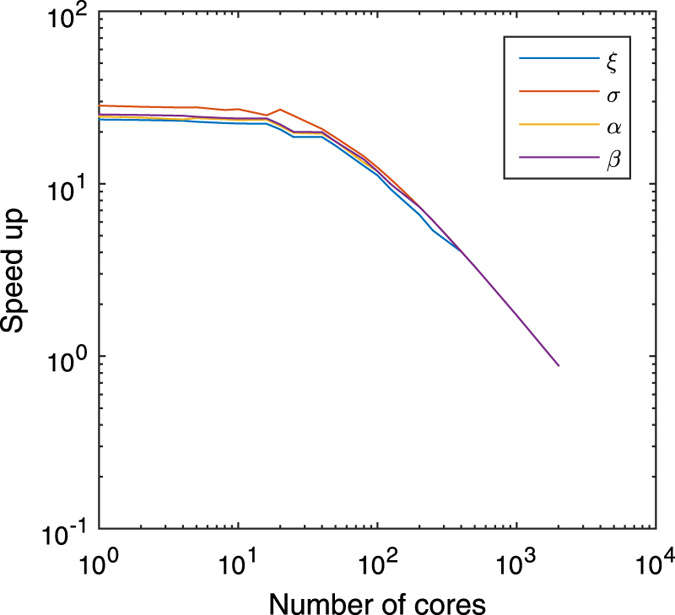
Speed up and parallelization. The speed up of the 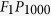 variant for the GEV distribution bootstrap, while reaching a high accuracy of the 95% confidence interval. The speed up, shown on the vertical axis is recalculated in case the method is run on multiple cores (horizontal axis), as explained in the method section. The speed up decreases for all the variables, 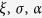 and 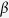 of the GEV model if more cores are used. If less than 20 cores are used, more than 90% of the speed up is preserved.

Resampling methods earn part of their popularity by the fact they can be computed easily in parallel. Note that this parallel computing is no problem for our speed up. Only if literally every resampled dataset is handled simultaneously on a different computational entity, our speed increase will vanish. As long as parts of the resampling method stay serial, one can learn from previous information and consequently enjoy a speed increase. This is clearly shown in Fig. 5 for the GEV example. Here we show the speed up factor of the 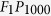 variant while reaching a high accuracy. When everything is run on 1 core, we have a speed up of about 25 for every parameter. When more and more cores are used, the speed up decreases. When 2000 cores are used, every core computes one bootstrap. In this case, nothing can be learned from previous bootstrap computations. The speed up factor is now even below one, our method is slower as the normal method as the fingerprint resampling needs time to compute the fingerprints. If 5 cores are used (normal desktop computer), the method still enjoys 97% of the maximum speed up. When 20 cores are used, as is possible on a very high-end scientific computer, the method can reach 90% of the maximum speed up. On a supercomputer, using 100 cores, 47% of the speed up is preserved.

## Discussion

The computational burden of resampling methods is oftentimes very large. Clearly, our fingerprint resampling method can decrease the computational cost enormously. The starting point of the fingerprint resampling is that in a resampling scheme, similar computations are done over and over again for very similar data. Ignoring the previous calculations would be a waste of time. By interpolating between structured previous information, predicting future optima and omitting the optimization process, we succeed in increasing the speed approximately by a factor of 10. We have illustrated our method with two simple examples but also with two real-life applications. The bootstrap of the mixed model can now be finished during a coffee break (20 minutes), while it took half a work day before (more than 4 hours).

In this paper, our method is proposed in such a way that it can be used for a broad range of resampling techniques. It can however easily be adapted to create a custom-made solution, increasing its effectiveness for more specific problems. For example, if the 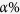 percentile has to be computed with a bootstrap, one can actually use a more advantageous, but more specific scheme. One can predict which resampled data sets are included in the <2*α*% interval with fingerprints and then use an optimization algorithm to find the real optima of these data sets. Using such a scheme, there is almost no error introduced while retaining the computational advantage. In another example, a Newton-Raphson optimization may be used. In such a case our method can easily be combined with 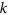-step method of Davidson *et al.*^21^. One can first do a raw prediction of the optimum and can then refine this optimum with 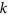 optimization steps.

Resampling methods earn part of their popularity by the fact they can be computed easily in parallel. Note that this parallel computing is no problem for our speed up. Only if literally every resampled dataset is handled simultaneously on a different computational entity, our speed increase will vanish. As long as parts of the resampling method stay serial, one can learn from previous information and consequently enjoy a speed increase.

Not all aspects of our fingerprint resampling method are known at this point. We discuss two of them. First, in the simulations of this paper, we treated all problems as local optimization problems. Problems with multiple local minima form however an even greater computational problem. Therefore we will address this problem in future research. Fingerprints may still be able to point in the right direction of the optima. The relation between the fingerprints and optima may however become very unsmooth if different resampled data sets converge to other kinds of minima.

Second, the final goal of most resampling techniques is to compute an uncertainty measure. It stays however unclear how much error is introduced in this uncertainty measure by predicting a certain amount of optima (the ratio 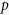). Most black-box techniques can provide reliable prediction errors of the optima themselves, but it can be hard to translate those prediction errors to errors in the uncertainty measure. On the other hand, this is not a problem specific to our method. When our speed up is not used whatsoever, one still has to assign the accuracy of the optimization for every resampled data set. Moreover, most resampling techniques introduce errors themselves. For example, one can never exactly know the error that is introduced by only computing a limited number of bootstraps^29^. Future research has to assess how all these errors are combined in the final uncertainty measure.

In conclusion, the fingerprint resampling method presented in this paper may fuel a more widespread use of resampling methods for complex models, which is now impossible or difficult because of the large computational burden.

## Methods

### A general description of the fingerprint resampling method

The basic idea of our method was explained in the result section. In this section we will develop our method in a more systematical fashion and indicate that it can be used more generally. For simplicity and completeness, we will consider the same four options as in the result section. However, only the third and fourth options are variants of our fingerprint resampling method.

#### Option 1: A naive starting value

Starting values are very important for optimization algorithms. They greatly define the speed in which the optimization process converges. In practice, when little information is known about the estimation process, naive starting values can be used (such as zero, one, the unity matrix, etc.). Such a naive starting value may be biased in the sense that it is systematically too high or too low compared to the real optima of the resampled data sets.

#### Option 2: The original optimum 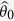 as a starting point

Taking the original optimum, 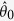, may lead to a serious improvement over a naive starting value. As the original data set is central to the resampled data sets, the original optimum is likely to be central compared to the optima of the resampled data sets.

In the context of bootstrapping, the estimated bias on an estimator 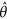 is calculated by




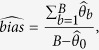




with 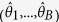 the 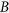 optima of the resampled data sets^29^. Assuming this estimated bias is zero, one has




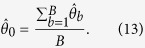




This means that the original optimum is exactly the mean of the bootstrap optima. As a consequence, if the estimated bias of the estimator 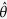 is zero, the bias of 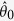 as a predictor for the optima of the resampled data sets, is also zero. Therefore, when no further information is known about a problem, this original optimum is usually a reasonably good initial value for the subsequent optimizations and it is easy to implement. Using the original optimum as initial value is the most unsophisticated way of using previous information and it is not unique to our procedure^19^,^20^,^21^. It may already lead to a significant speed increase as shown in the result section.

#### Option 3: Learn from the previously resampled data sets

In a next phase we will try to create better predictions for the optima of a new resampled data set making use of the previously resampled data and their corresponding optima. The better the prediction, the better the starting value and the less iterations the optimization procedure needs. For this prediction, we need two ingredients. A first ingredient is a concise summary of the data sets into statistics, called fingerprints, which can be linked to the optima of the resampled data. As a second ingredient, we need a method that can be used to learn the relation between the fingerprints and the optima of past data sets.

It is clear that, in order for our method to be efficient, both the fingerprints and the method to learn the relation should place a minimal computational burden on the whole resampling calculation. In the following parts, we will discuss both ingredients. To conclude, we discuss the link with the tuning parameters of the optimization algorithm.

### Fingerprints

A fingerprint 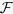 is a scalar-valued or vector-valued function taking the resampled data set as input and produces one or more statistics as output. (In what follows, we will denote both the function as well as the function values with the term fingerprint.) To use such a fingerprint for the prediction of the optimum, it must contain information on this optimum in a parsimonious manner. Obviously, there is a trade-off between compression rate and retained information. For instance, a trivial fingerprint would be the identity function, which maps a data set onto itself. Such a fingerprint obviously contains all the necessary information to locate the optimum. However, the relation between the fingerprints and the optima is harder to derive when the fingerprint has a high dimensionality. Therefore, the identity function does not qualify as a good fingerprint because there is no information reduction.

The question then becomes what constitutes a good fingerprint. A derivative-based fingerprint is intuitively attractive. The rationale behind a derivative-based fingerprint is that the information contained in derivatives (rate of change, curvature, etc.) in a point near a local optimum will tell us something about the location of the local optimum itself. In fact, the same kind of information is used in several optimization algorithms (e.g., steepest descent and Newton-Raphson). Therefore, we propose to use the derivatives of the objective function of the resampled data set, evaluated in the original optimum, as a fingerprint. Formally, this can be written as follows in the case of maximum likelihood estimation (for a particular resampled data set 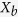):




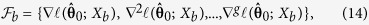




where 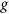 is the order up to which the partial derivatives are used. (In case of least squares estimation 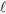 can be replaced by a sum of squares loss function 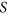.) Of course, only the unique elements of the partial derivatives are used for the fingerprint: For example, if 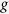 equals 2, these are the first order derivatives combined with the upper triangular elements of the Hessian matrix.

The derivatives can be approximated using finite differences if no analytical expressions exist. Obviously, a fingerprint using finite differences gives another incentive to reduce the dimensionality of the fingerprint. The higher the order of the derivative that needs to be estimated, the more function evaluations need to be computed. If 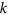 is the dimension of the optimum, one needs at least 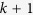 function evaluations if 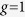 and 
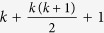
 function evaluations if 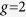 when finite differences are used.

A more fundamental reason to use derivative-based fingerprints is provided by Taylor’s theorem. If a function can be completely approximated by a Taylor series, all its information can be summarized in its derivatives evaluated in a certain point (e.g., the original optimum). Taking an infinite number of (partial) derivatives (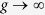) will then lead to a fingerprint with all information needed to uniquely define the optimum of its function. However, it is not necessary to describe the function one wants to optimize, nor its associated data set in statistics. Already in a very compact fingerprint a lot of information can be found about the optimum. As already mentioned, we propose to take a fingerprint with a dimension as low as possible, for modeling reasons. A limited number of derivatives, for example all first-order derivatives in combination with all the unique elements of the Hessian matrix, will suffice in many cases.

It should be noted that a good fingerprint for one model does not necessarily generalize to a good one in another model. Sometimes, the likelihood cannot be evaluated in the original optimum for some data sets (see e.g., the example on the generalized extreme value distribution in the method section), making it impossible to compute the derivatives. Moreover, other ways of summarizing data may work at least as good as the derivative-based fingerprints. For example, computing the moments of a data set can also lead to good results. The sample average in the first example on the exponential distribution would obviously be a good fingerprint. Another possibility is to use summary statistics as they are used in approximate Bayesian computing. It is even possible to automatically generate such summary statistics^42^. In any case, what constitutes a good fingerprint is very problem specific. A covariance is decisive for the optimum in a regression while it cannot be computed in the data set of an exponential distribution. In situations where one has specific prior knowledge about the model, one can use this information to create a better model-specific fingerprint.

#### Predicting initial values based on fingerprints

Once the fingerprints are computed, the relation between the fingerprints and corresponding optima needs to be modeled. This relation will then be used to predict the optima of subsequent resampled data sets. These predicted optima can then be used as starting points.

The modeling of the relation between fingerprints and optima should satisfy three requirements. First, the modeling should be fast to calculate. The main reason for the prediction of the optima and using these as starting values is to create a significant speed up in the optimization procedure. However, this gain would be wasted in case of a slow modeling procedure. Second, the modeling should be able to describe a nonlinear relation. One cannot expect to only find strictly linear relations between the fingerprints and the optima. Already in the second simple example in the result section (on regression), the relation between optima and fingerprints is nonlinear. Third, we prefer to choose a method which excels in prediction. Both support vector machines (SVM^43^) and multivariate adaptive regression splines (MARS^28^) are well known for their generalization quality, which lead to good predictions. For the SVM, we propose to use a specific adaptation, the least squares support vector machines (LS-SVM), which lead to a much faster modeling compared to traditional SVM^26^. Note that these methods create a multi-input single-output model. This means that one needs one model for every dimension of the parameter that needs to be optimized. Because every dimension of the fingerprint initially has the same importance regarding the predictions, they are all standardized to train the black-box model.

The black-box methods are versatile, flexible and fast to compute. However, there are two specific problems that need to be addressed before the methods can be applied efficiently: constraints and extrapolation.

**Figure 6:**
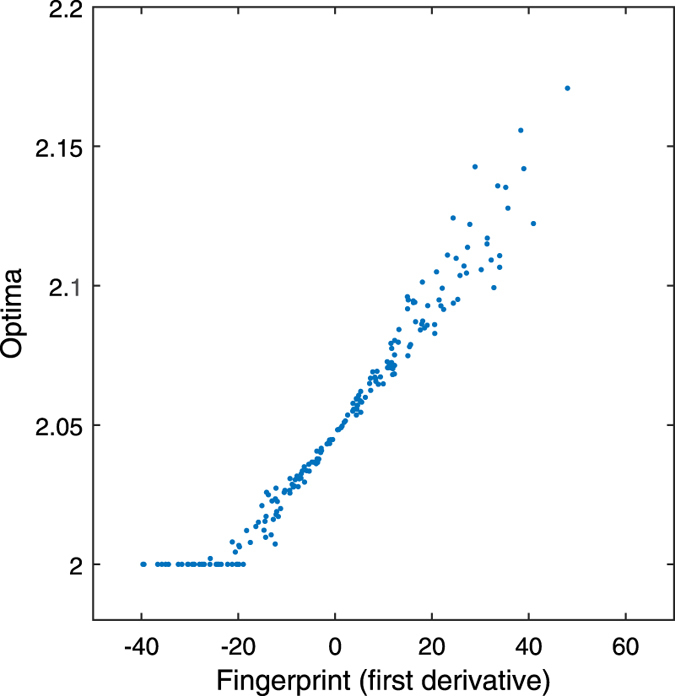
Fingerprints and optima of a constrained linear regression. A linear regression data set with estimated slope (the original optimum) 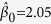, is non-parametrically resampled 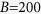 times. All the optima are found with a constrained optimization, it is assumed that 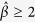. The horizontal axis shows the fingerprints 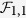 to 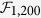 and the vertical axis the optima 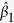 to 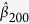.

A first problem that needs to be solved is that some estimation problems call for a constrained optimization. If a parameter is constrained, this can lead to a non-smooth fingerprint-optimum relation. As an illustration, consider Fig. 6 which can be compared to Fig. 3d in the result section. In both figures the fingerprints of a linear regression are plotted against optima. However, in Fig. 6, the optima are found with a constrained optimization (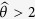). A situation is shown where the fingerprints below a value of -30 all result in a value of 2 of the parameter 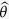. It can be seen that if the constraint is active, then for multiple resampled data sets, different fingerprints will all lead to the same optima, 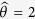. When at the same time, other resampled data sets lead to an inactive constraint, those resampled data sets will lead to a different fingerprint optima relation. There are now two different relations between the fingerprints and the optima. As a result, the relation between fingerprints and the optima is non-smooth. A black-box technique may have a hard time to learn such a non-smooth relation. For such cases, we propose the following solution. First, the black-box technique is only be estimated with the optima which are not on the boundary. Second, if the black-box technique makes a prediction that violates the constraint, the prediction is adapted to the constraint value.

A second problem is that we need to avoid extrapolation and the black-box methods can only be used to interpolate. However, because we learn sequentially from the resampled data, it is very likely that we encounter for resampled data 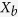 a fingerprint value 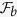 that lies outside the range of previously modeled data. To circumvent the extrapolation, we propose the following course of action during the initial phase. First, the fingerprints of all resampled data sets are computed. Then, for each dimension of the fingerprint, the minimum and maximum is searched. For the data sets corresponding with one or more extrema, the optima are computed first. In total, 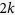 data sets are prioritized, with 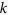 the dimension of the fingerprint. Second, a fair number of purely random data sets (usually, 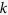) are added and also for these data sets the optima are computed. Postponing the use of the predictions for these initial data sets ensures a stable model over a broad range of fingerprints. To find the optimum, the original optimum is used as a starting value in this initial phase.

#### Other tuning parameters

The speed of most optimization algorithms can be improved and controlled by tuning parameters (e.g., the initial step length in line search algorithms for gradient-based methods; the size of the initial simplex in the Nelder-Mead method). It can also be useful to control these tuning parameters based on previous optimizations. Important in this control problem is that the uncertainty associated with the starting value should not be neglected. If the starting value is of high quality (because the predicted optimum and the true optimum are close together), the optimization algorithm should initially only search for an optimum in a small area around it. Otherwise, the advantage of a good starting value may disappear. The quality of the starting value can be extracted from the prediction error of the interpolator.

#### Option 4: Bypassing the optimization altogether

If more and more optima are computed, more and more information becomes available to train and improve the model that estimates the relation between the fingerprints and the corresponding optima. If both the fingerprints and the method to model this relation are chosen well, prediction errors of subsequent optima can become very small. When these predicted optima are used as starting values, the optimization process will only add little accuracy to the estimated optima. The starting values can become practically indistinguishable from the real optima. In such a case, it is not a problem to bypass the optimization process because the accuracy that can be gained is negligible compared to intrinsic (i.e., Monte Carlo) error in the resampling itself^1^,^21^. Therefore, it is unnecessary to know the optima with highest possible precision. The predicted optima, including a small error, can just as well be used as the real optima. This brings the speed increase of our method to a whole new level, the optimization process can now be skipped. As we note in the discussion, it can be hard to estimate how this prediction error exactly influences the accuracy of the uncertainty measure the resampling method computes.

### Assessing the performance of the methods

The performance of the methods is assessed from two perspectives. First, the time needed to estimate the parameters for the series of resampled data sets (averaged over a number of replications in order to reduce the error) is evaluated. Second, we assess how the different methods deal with an intrinsic speed-accuracy trade-off: A longer computation time leads to more accurate results, but how does the accuracy of the methods compare given a fixed computational load?

We measure computation time in terms of number of function evaluations (where the function is the objective function that needs to be optimized for every resampled data set, such as the likelihood or least squares loss function). The two computationally most expensive parts of our method (i.e., the time the optimization algorithm needs to converge and the computation time of the derivative-based fingerprints) can be measured in this number of function evaluations.

The method proposed in this paper will be most useful if the optimization process is unbearably slow. Another aspect of our method involves the modeling of the relation between the fingerprints and the optima and does not place a large burden on the computation. It can be neglected because it does not scale with the difficulty of the optimization process. The relative amount of time needed for the interpolator (such as the LS-SVM or MARS) will be close to zero in cases of high computational burden. For example, using one core on an Intel Core i7-3770 with 3.4 GHz, the execution time of the estimation of one LS-SVM is 1.5 seconds with a training set of 200 previous fingerprints and optima.

Another reason not to consider the interpolator in the total computational cost is because it is unnecessary to update the modeled relation between fingerprints and optima after each optimization process. The modeled relation and the quality of the predictions will only significantly change if the training data changes significantly. This will not be the case if only one couple (i.e., fingerprint and associated optimum) is added. We propose to update and remodel the relation only when 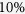 more information is available. In practice, the modeling of the relation can be stopped if the reduction in prediction error is only marginal. We stop this modeling when more than 1000 optima are found. Other machine learning improvements are possible to further reduce the time needed by this modeling, like online updating and big data solutions^27^. All these options are however not included to present a much cleaner picture of our method.

In sum, when considering computational time, we opt to neglect the part spent to modeling the relation between fingerprints and optima in the determination of computational time and only express the computation time in number of function evaluations. This gives the opportunity to also study the effects of our method in simpler problems, where the optimization process is very fast (such as for the two simple examples).

### Optimization algorithm

The optimization procedure used by fingerprint resampling is not central to the method. In the result section we use the Nelder-Mead method^44^. This algorithm is used for its generality and its popularity^45^. It is derivative free and can be used on almost any convex function. Moreover, it is the default algorithm used by the basic optimizers in Matlab and R (i.e., 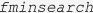 in Matlab^46^,^47^, and 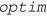 in R^48^) and it is used in the popular 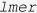 function in R to estimate mixed models. The Matlab function 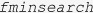 is used as basic implementation of this algorithm.

The Nelder-Mead method is an optimization algorithm that uses simplexes with 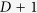 vertices 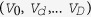 , with 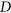 the dimension of the parameter vector to optimize. When initializing the algorithm, vertex 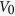 is set to the starting value, 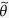. For example, in the 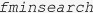 function, the complete initial simplex is then assigned similar as in Algorithm 1.




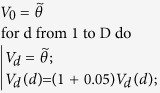




#### Algorithm 1

Initialization of the initial simplex as done by 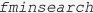, for 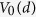 not equal to zero. 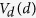 is the 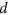-th entry of vector 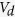.

The initial simplex can be improved upon considerably when more information becomes available. When modeling the relation between the fingerprints and the optima, future optima are predicted but we can also estimate an error of these predictions. For instance, this error can be estimated with a fast leave-one-out cross-validation. The interpolator will assign for dimension 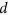 of the parameter vector a prediction error, denoted 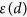. The assignment of the initial simplex is then changed as indicated in Algorithm 2.




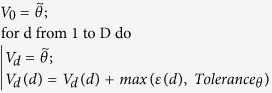




#### Algorithm 2

Initialization of the initial simplex using the prediction error 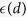 for each dimension 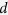. 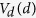 is the 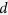-th entry of vector 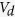. 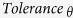 is the user-defined value for assessing convergence based on the parameter values.

The convergence criterion used for the Nelder-Mead optimization method is when the maximum distance (infinite norm) between the vertices is smaller than 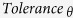 and the maximum difference between their corresponding function values is smaller than 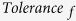. In our applications, both tolerances are set to 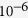.

### Mathematical models used

In the result section we make use of an exponential distribution, a linear regression, a mixed effects model and a generalized extreme value distribution. The mathematical model of the exponential distribution is already discussed in this result section. Here we will give every necessary detail about the other three models.

#### Linear regression

In the second simple example in the result section, a linear regression model is used. With data set 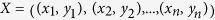, regression weight 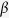 and known variance 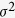, its likelihood is given by




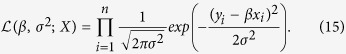




Its loglikelihood is given by




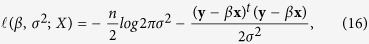




with 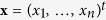 and 
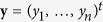
. For simplicity, only the case where 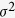 is known and equal to one is considered. The loglikelihood simplifies to




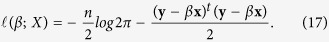




In the result section, 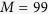 synthetic data sets are simulated with 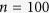, 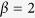 and 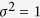. For each simulated data set, 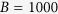 bootstrap data sets are generated. For each bootstrap sample this loglikelihood is maximized for the single parameter 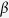. Its analytical solution is given by




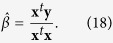




As explained in the result section, an iterative optimization algorithm is used to optimize the loglikelihood.

For variant 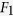, a one-dimensional fingerprint is created using the first derivative of the loglikelihood in the original optimum:




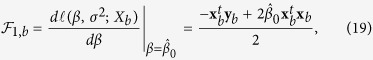




For variant 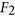, a two-dimensional fingerprint is created by combining this fingerprint with the second order derivative of the loglikelihood in the original optimum:




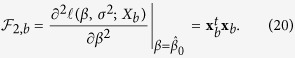




Both derivatives are computed with finite differences in the result section. As shown in the result section, one cannot create an exact function between fingerprint 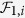 from Equation 19 and the optimum 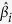 from Equation 18 (variant 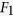). However, using a two dimensional fingerprint (variant 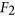), using Equations 18, 19 and 20 one can derive the following functional relation:




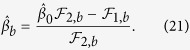




This relation leads to much better predictions of new optima, as is illustrated in the result section.

### Mixed model

In line with Verbeke & Molenberghs^8^, we start with a random sample of 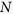 units. For each unit 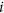, 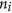 measurements are taken with respect to an outcome variable and these measurements are collected in a vector 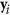. Next, the following model is assumed:




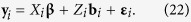




with 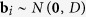 and 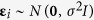 and 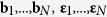 independent. The parameter vector 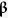 contains the fixed effects regression coefficients and 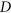 is the population variance-covariance matrix. Such a general linear mixed model implies the following marginal model:




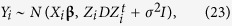




The loglikelihood is then given by




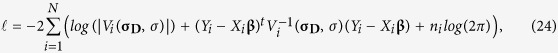




with




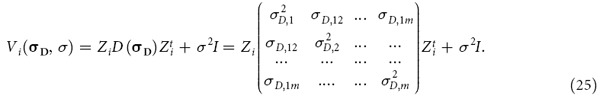




and 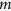 the number of random effects. In practice however, it is better to use a differently parametrized loglikelihood^49^,^50^. First, to assure the positive definiteness of this matrix, we use a Cholesky decomposition of 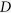
49. Second, to reduce the number of parameters of the optimization problem, 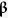 and 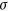 can be profiled out^50^. This leaves us with unique 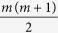 parameters, 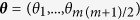, to optimize. To find the variances of random effects of the mixed model, the resulting optima (both the ones found after an iterative optimization and the ones predicted) have to be translated back to their original parametrization, as used in Equation 24. Note that, due to the Cholesky decomposition, some of these parameters can only have a positive contribution in Equation 24. For example, a parameter 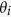 is only used as 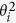, even when 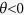 it can only have a positive contribution 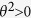. This means that we introduce an indirect constraint.

In the data of Bringmann *et al.*, used in the result section, both the intercept and the slope are assumed to vary across patients^14^. No restrictions are imposed on the variance matrix of these random effects (except for positive definiteness):




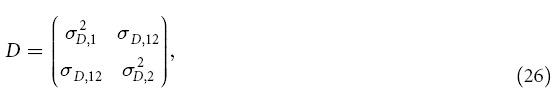




which leads to a three dimensional optimization problem.

To bootstrap this data, we use the so-called case bootstrap^30^,^31^. The case bootstrap is a non-parametric bootstrap which uses the clusters (in our case, the patients), as entities to sample from. The whole bootstrap process is repeated 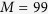 times, each time with 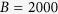 new random bootstrap iterations.

In the software package (see the Code availability paragraph), in the exampleFingerprintBootstrap.m file, the fingerprint bootstrap is also applied on a mixed model using artificial data.

### Generalized extreme value distribution

For the generalized extreme value distribution with data set


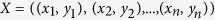
, shape parameter 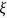, scale parameter 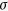 and location parameter 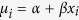, the likelihood is given by




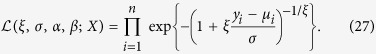




In the data set used in the result section, 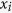 is given by the maximum windspeed of a certain day, and 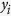 is given by the date (number of days after first measurement). The logarithm of this likelihood is computed using an adapted version of gevlik.m of Matlab^46^. To find the maximum of this loglikelihood, a four dimensional optimization problem is solved using the Nelder-Mead method. As a naive starting value we use moment estimators as proposed by Hosking *et al.*^51^ and a least squares estimation for the 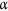 and 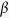 parameters^52^.

It is interesting to note that the likelihood can only be computed if 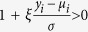. It is therefore possible that for some data sets, the fingerprint resampling method might need to be adapted. If the original optimum leads to an invalid likelihood for some bootstrap data sets, the original optimum cannot be used as a starting value, nor can the derivatives be calculated to create a fingerprint. In such a case, it is advised to use another sort of fingerprint. For the data set in the result section, there were no such problems.

### Speed-accuracy trade-off for the bootstrap

In the result section, we estimate the lower bound of a 95% confidence interval using bootstrap. From a series of 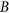 bootstrap samples, this lower bound is the 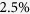 percentile, denoted as 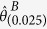. Using the percentile bootstrap confidence interval method, this percentile can be estimated as^29^:




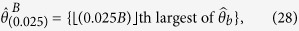




where 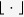 is the flooring function. Obviously, it is always better to use more than less bootstrap iterations. For the mixed model problem we use 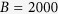 bootstrap iterations as the end goal. However, we also monitor 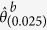 as a function of 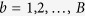. For any 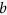 or 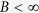, 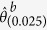 and 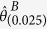 will always be approximations of 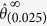, which uses an infinite number of bootstrap iterations. Adding bootstrap optima one after the other, our estimate 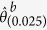 will come closer and closer to 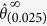. The error will decrease further and further. However, more bootstrap iterations generally means more function evaluations. Hence, there is an intrinsic trade-off between speed (number of function evaluations) and accuracy (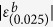, the absolute difference between 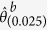 and 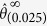). 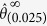 itself can obviously not be computed. For the bootstrap of the mixed model, it is approximated using 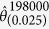, combining all bootstrap iterations of the 99 bootstrap processes. This combination of all bootstrap iterations over all the bootstrap processes is allowed, as each bootstrap process is created using the same original data set and all bootstrap data sets are generated independent and at random. The accuracy can now be computed as:




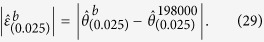




The exact speed up our method achieves is measured at two different accuracies. First, it is measured at what is called a high accuracy. This is approximately the accuracy of the original optimum variant when the bootstrap process is completed, after 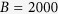 bootstrap iterations, 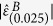. Second, it is measured at what is called a medium accuracy, which has an error twice as big as the high accuracy: 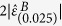.

As explained before, resampled data sets with extreme fingerprints are prioritized (to avoid extrapolation). This means that potentially extreme optima are found first which may disturb the accuracy of 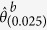 for small 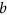. We controlled in an ad hoc way for this artefact by assigning weights to these prioritized optima related to the inverse of the original index of their associated data set. Resampled data sets that are prioritized will get a lower weight for small 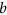. Note that this correction has no effect at all if all optima (predicted or found by an optimization process) are used. So in a normal implementation, where all optima will always be used, this correction is of no importance. Only for the speed-accuracy trade-off figure (to show the evolution of the accuracy), such a correction must be made.

To estimate the effects of parallelization for a set up with 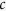 cores, the function evaluations and accuracies are grouped together in batches of 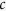 bootstrap estimations. The whole batch would be computed simultaneously on the 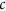 cores. In a parallelization context, bootstrap estimations which are in the same batch cannot learn from each other, only information of bootstrap estimates of previous batches is available. The speed up is recalculated undoing the effects of this information which is now unavailable. For example, if 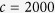 cores are used to compute 2000 bootstrap estimates, all estimations happen in the same batch. Therefore, since no bootstrap estimation can be speeded up using information of any previous calculations, the fingerprint resampling method would be useless in such a case.

### Code availability

Our method is implemented in Matlab, the software package and all data necessary to replicate this paper can be found at

http://ppw.kuleuven.be/okp/software/fingerprintresampling/. The original data set used for the mixed model example can also be found at

http://journals.plos.org/plosone/article?id=10.1371/journal.pone.0060188^14^. The original data set used for the GEV example can be found at

http://www.knmi.nl/climatology/daily_data/selection.cgi^41^. Most results were obtained using Matlab 2014a on a supercomputer using 5 nodes (100 processors)^46^. One processor was used to coordinate the 99 other processors. Each processor was used to compute one bootstrap process of 2000 bootstraps, using all possible variants. This is the reason why exactly 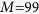 bootstrap processes are used to illustrate the speed up of our method.

## Additional Information

**How to cite this article**: Mestdagh, M. *et al.* Fingerprint resampling: A generic method for efficient resampling.. *Sci. Rep.* **5**, 16970; doi: 10.1038/srep16970 (2015).

## Figures and Tables

**Table 1:** Overview of all the variants.

Option	Name	Abbreviations	Short explanation
1	Naive starting value		An arbitrary starting value.
2	Original optimum		Use the optimum of the original data set as starting value.
3	Learn from the previously resampled data sets	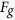 or 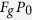	Use previous information to predict future optima. Use these predictions as starting values. Fingerprints are used to summarize previous information. The index 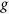 means that partial derivatives of the loglikelihood function up to order 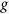 are used to construct the fingerprint.
4	Bypassing the optimization altogether	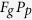	Use previous information to predict future optima. Use these predicted optima in the resampling method. Fingerprints are used to summarize previous information. The index g means that partial derivatives of the loglikelihood function up to order g are used to construct the fingerprint. The number p refers to the ratio between the number of resampled data sets for which the optimization is bypassed compared to the number of resampled data sets for which the optimization algorithm is fully used.

**Table 2:** Speed up results for the bootstrap of a mixed model.

Parameter	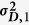		σ_D,12_		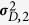	
Accuracy	medium	high	medium	high	medium	high
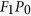	1.4	1.4	1.3	1.3	1.2	1.4
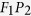	3.7	3.9	3.0	3.8	3.8	4.2
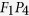	5.7	5.9	4.1	5.8	5.3	5.3
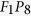	8.1	9.0	5.6	8.5	6.5	4.8
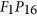	13	12	6.3	9.0	9.1	4.8
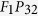	17	12	7.9	9.0	9.1	4.8
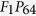	14	12	6.8	9.0	7.2	4.8
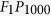	6.0	12	4.2	9.0	4.3	4.8
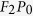	1.4	1.7	1.4	1.7	1.3	1.8
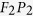	3.0	3.7	2.9	3.7	2.8	3.9
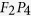	4.1	5.0	3.8	4.9	3.7	5.3
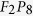	5.4	6.5	4.9	7.0	4.8	7.1
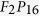	6.8	7.9	4.5	8.6	5.6	8.7
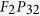	4.9	9.0	3.5	8.5	3.7	6.3
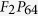	2.9	8.9	2.0	8.1	2.2	6.3
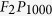	2.6	8.9	1.9	8.1	2.0	6.3

The table shows how much faster the different variants, 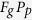, get at a high accuracy (approximately the lowest error achieved by the original optimum variant) and a medium accuracy (approximately twice this error). These accuracies are shown in Fig. 4. Results are shown for the 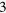 parameters of 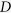: 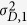, σ_D,12_ and 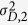. The 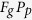 variants are up to 17 times faster as the original optimum variant. More generally, for all the model parameters over the majority of the accuracies, one can always find a 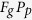 variant that leads to an 8 to 17 times faster bootstrap compared to the original optimum variant.

**Table 3:** Speed up results for the bootstrap of a GEV distribution.

Parameter	ξ		σ		*α*		*β*	
Accuracy	medium	high	medium	high	medium	high	medium	high
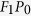	1.6	1.6	1.3	1.7	1.6	1.7	1.6	1.7
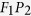	4.2	4.6	3.5	4.2	4.2	4.6	4.3	4.6
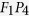	6.3	6.7	5.2	6.9	6.1	7	6.3	7
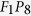	9.4	10	7.8	4.8	9	11	9.4	11
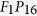	14	15	11	4.8	13	16	13	16
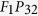	17	20	13	21	16	21	11	21
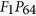	17	24	10	26	13	26	9.1	25
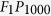	9.4	24	5.7	28	8.9	25	6.4	25
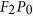	1.9	2.3	1.5	2.4	2	2.4	1.9	2.4
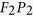	3.7	4.6	2.7	4.8	3.7	4.8	3.5	4.9
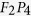	4.6	5.7	3.4	6	4.6	6.2	4.4	6.2
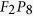	5.9	7.2	4.3	7.7	5.7	7.7	5.6	7.8
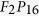	6.5	8.5	3.9	9.3	6.1	9.1	4.7	9.3
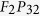	4.3	9.3	2.6	10	4.1	10	3.2	11
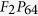	3.4	9.6	2	11	3.2	10	2.5	10
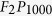	3.4	9.6	2	11	3.2	10	2.5	10

The table shows how much faster the different variants, 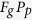, get at a high accuracy (approximately the lowest error achieved by the original optimum variant) and a medium accuracy (approximately twice this error). Results are shown for the 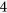 parameters of the GEV: 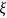, 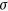, 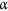 and 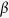. The 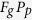 variants are up to 28 times faster as the original optimum variant. More generally, for all the model parameters over the majority of the accuracies, one can always find a 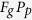 variant that leads to a 24 times faster bootstrap, compared to the original optimum variant.
